# Preliminary genetic analyses of important musculoskeletal conditions of Thoroughbred racehorses in Hong Kong^☆^

**DOI:** 10.1016/j.tvjl.2013.05.002

**Published:** 2013-12

**Authors:** Claire E. Welsh, Thomas W. Lewis, Sarah C. Blott, Dominic J. Mellor, Kenneth H. Lam, Brian D. Stewart, Timothy D.H. Parkin

**Affiliations:** a School of Veterinary Medicine, University of Glasgow, 464 Bearsden Road, Glasgow, Scotland G61 1QH, United Kingdom; b Animal Health Trust, Newmarket, England CB8 7UU, United Kingdom; c Sha Tin Racecourse, Hong Kong Jockey Club, Department of Veterinary Regulation and Internal Liaison, Sha Tin, Hong Kong, China

**Keywords:** Fracture, Heritability, Thoroughbred, Musculoskeletal

## Abstract

A retrospective cohort study of important musculoskeletal conditions of Thoroughbred racehorses was conducted using health records generated over a 15 year period (*n* = 5062, 1296 sires). The prevalence of each condition in the study population was: fracture, 13%; osteoarthritis, 10%; suspensory ligament injury, 10%; and tendon injury, 19%. Linear and logistic sire and animal regression models were built to describe the binary occurrence of these musculoskeletal conditions, and to evaluate the significance of possible environmental risk factors. The heritability of each condition was estimated using residual maximum likelihood (REML). Bivariate mixed models were used to generate estimates of genetic correlations between each pair of conditions.

Heritability estimates of fracture, osteoarthritis, suspensory ligament and tendon injury were small to moderate (range: 0.01–0.20). Fracture was found to be positively genetically correlated with both osteoarthritis and suspensory ligament injury. These results suggest that there is a significant genetic component involved in the risk of the studied conditions. Due to positive genetic correlations, a reduction in prevalence of one of the correlated conditions may effect a reduction in risk of the other condition.

## Introduction

Musculoskeletal conditions and injuries (MSC) are commonly encountered in Thoroughbred racehorses worldwide. Their prevalence tends to exceed that of cardiac problems and epistaxis, which contribute significantly to the burden of health problems for Thoroughbred racehorses (Williams et al., 2001). The frequency and severity of MSC create a substantial financial burden for the racing industry, compromise equine welfare and create negative publicity for the sport. Many previous studies have attempted to identify risk factors for MSC in racehorses (see, for example, Stephen et al., 2003; Ely et al., 2004; Parkin et al., 2004a; Pinchbeck et al., 2004; Jacklin and Wright, 2012). These studies varied in many ways, including case definition, identity of the target population, and whether diagnoses were made in racing and/or training.

Due to differences in the spectra of risk factor associations, and in the populations to which those findings can be extrapolated, robust advice for stakeholders on the avoidance of risk of MSC has not been forthcoming. Few previous studies have attempted to identify a significant role for genetic risk in the development of these conditions, and have focused Warmblood breeds rather than Thoroughbreds (Pieramati et al., 2003; Oki et al., 2008; Jonsson et al., 2011). The identification of a significant genetic component to disease aetiology could be exploited as a basis for controlling risk in conjunction with the modification of environmental factors. The ultimate goal in this pursuit is to minimise individual and population risks through the prudent use of both environmental and genetic disease information. In this study, we estimated the heritability of a number of important MSC in racing Thoroughbreds in Hong Kong, to provide a benchmark for subsequent genetic analyses.

## Materials and methods

### Data

The data used in this study were provided by the Hong Kong Jockey Club (HKJC). All Thoroughbred racehorses in Hong Kong are owned by HKJC, and are housed together at Sha Tin Racecourse in the New Territories, and managed as a unit. As there are no racehorse breeding facilities in Hong Kong, all HKJC Thoroughbreds are imported. All racing is on two flat courses, Happy Valley in Hong Kong city and at Sha Tin. Racing surfaces are composed of all-weather ‘dirt’ or sand based turf.

**Fig. 1:**
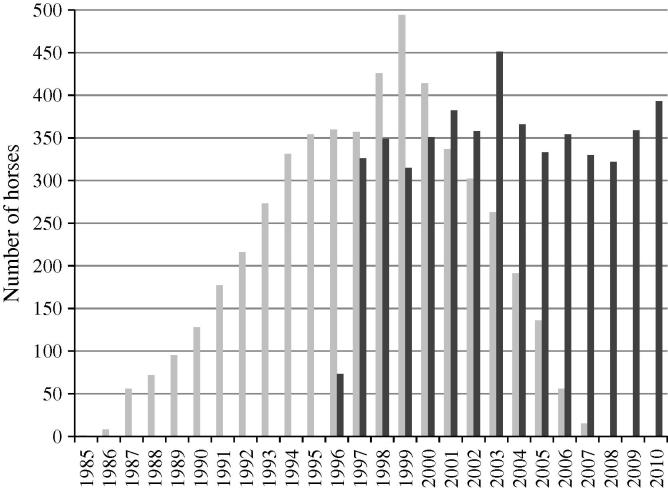
Number of horses in the current dataset that were born or retired between 1985 and 2010. Light grey bars, year of birth; dark grey bars, year of retirement.

The HKJC employs a team of full-time veterinarians who are responsible for the clinical care of all horses. Compulsory retirement is enforced for horses that reach 10 years old, have two or more officially recorded episodes of exercise-induced pulmonary haemorrhage or have two occurrences of cardiac arrhythmia. Retirement for other medical conditions, or for management reasons, is at the discretion of the veterinarian, trainer and owner, and can occur at any time. Horses are continually imported and retired throughout the racing year, with the racing season taking place between September and June. Fig. 1 contains information on the years of birth and retirement of these horses. The health and pedigree records used in this study were collected by the HKJC and stored in a purpose-built Microsoft Access database.

During the period in which the data for this study were collected there were a median of 1028 horses per annum in training. Horse health information was contained in two tables; one contained records of the results of all Official Veterinary Examinations (OVEs) carried out between 1995 and 2010 (8690 records), and the other contained free text entries detailing the reason(s) for retirement from racing of all horses retired between 1992 and 2010 (5520 records). An OVE may be requested by the trainer/owner/steward at any time, in response to detection of a possible veterinary problem. The horse must ‘pass’ an OVE before being permitted to continue racing. The results of these examinations are the content of the OVE records used here. The OVE and retirement tables were merged after indexing by alphanumeric horse identification codes and all records entered before September 1996 were removed due to a significant amount of incomplete information. Pedigree information included the name of the sire, dam, paternal and maternal grandsires for each horse. Missing or erroneous pedigree information was corrected using a Thoroughbred pedigree information site.^1^ The resulting composite table of 5062 complete records, detailing all OVEs and subsequent reasons for retirement, was uploaded into content analysis software WordStat v6.1 (Provalis Research).

Free text records containing disease information were evaluated for the presence of words or phrases indicating diagnosis of certain user-defined categories of disease (Lam et al., 2007). The presence or absence of each disease category (coded 1 or 0) in each horse was exported as a Microsoft Excel spreadsheet (thus multiple records per horse were condensed into a single record per horse). All text or alphanumeric variables were numerically recoded before heritability analyses were performed. Conditions investigated were fracture, sesamoid bone fracture, distal limb fracture (inclusive of carpus/tarsus), suspensory ligament injury, tendon injury, superficial digital flexor tendon injury, degenerative joint disease and osteoarthritis.

### Case definitions

A horse was defined as a case for a condition if it had ever been recorded as suffering from that condition, whether this was a cause of retirement or an OVE. Controls for each condition were horses that had never been diagnosed with that condition.

### Non-genetic effects

Some data were available on potential environmental risk factors at the horse level. The gender of the horse was categorized as male or female, with neuter status at the time of retirement coded as ‘0’ for not neutered and ‘1’ for neutered. Year of birth, retirement and importation to Hong Kong were recorded as four digit numbers, e.g. 1999. Age at retirement was recorded in years (range 2.2–10.9, mean 6.0). The age at the time of import to Hong Kong ranged from 1.7 to 7.0 years (mean 2.8 years).

The provenance of each horse was captured in a number of variables: country of origin of the horse, its sire, dam and maternal grandsire were recorded as ‘1’ for Australia or New Zealand, ‘2’ for the UK, Ireland or the USA, and ‘3’ for all other countries; the continent of origin was recorded as ‘1’ for Europe, ‘2’ for Australasia, ‘3’ for North America and ‘4’ for all others, and the hemisphere of origin was recorded as either Northern (1) or Southern (2).

The lifetime winnings of each horse were recorded in Hong Kong Dollars. The number of races run throughout the career of each horse was recorded as an integer, and the ‘length of career’ calculated as the difference in days between the first race date and the date of retirement. A variable called ‘intensity’ was created by the length of career in weeks, divided by the number of races run generating the mean number of weeks between adjacent races. A total of 15 variables were available for analysis.

### Model building

Summary statistics for each continuous variable were produced and assessed, and categorical variables were examined for sufficient distribution across levels. Where numbers of cases or controls within a level fell below a pre-defined minimum of five, amalgamation of a number of levels was considered. Univariable analyses were conducted to identify relationships between all potential risk factors with all conditions. Variables with *P*-values of <0.2 were retained for multivariable modelling. All retained variables were ordered by log likelihood before sequential insertion into each multivariable model. Each multivariable model included sire or animal as a random effect (i.e. each outcome was modelled twice) prior to the inclusion of fixed effects. Fixed effect variables were retained within a multivariable model if the Wald test *P*-value was <0.05.

Within each model, correlation coefficients between every pair of retained variables were produced, and any >0.8 were investigated for the effects of removal of one of the correlated variables. All potential two-way interactions were investigated and retained if the Wald *P*-values of the interaction term and the likelihood ratio test were <0.05. All model building was performed in R v.2.15.1 (R Development Core Team, 2008), using packages ‘lme4’ (Bates et al., 2012) and ‘MKmisc’ (Kohl, 2013).

### Heritability and genetic correlations

The final models were analysed using ASReml v.3 genetic analysis software (VSN International), and heritabilities were calculated from the variance components. Each condition was investigated using both sire and animal models, on linear and logistic scales. The general form of the linear model was:

Y=Xb+Za+e

where *Y* is the vector of observations, *X* and *Z* are known incidence matrices, *b* is the vector of fixed effects, *a* is the vector of random additive genetic effects with the distribution assumed to be multivariate normal with parameters ($0\text{,}\sigma_{s}^{2}I$) for sire models and ($0\text{,}\sigma_{a}^{2}A$) for animal models, *e* is the vector of residuals with multivariate normal distribution and parameters ($0\text{,}\sigma_{e}^{2}I$), and where I denotes an identity matrix, *A* is the numerator relationship matrix, and *σ*^2^ denotes variance. The general logistic model form was:

$$\log(\frac{p}{1-p})=\mathrm{Xb}+\mathrm{Za}+e$$

where *p* denotes the prevalence of the condition in the population, and all other components are as before. Residual variance in logistic models was set to $\frac{\pi^{2}}{3}$. Heritability in sire models was determined by:

$$h^{2}=\frac{\sigma_{s}^{2}\times 4}{\sigma_{p}^{2}}$$

where $\sigma_{s}^{2}$ is sire variance, and $\sigma_{p}^{2}$ is total phenotypic variance, composed of sire and residual variance. Animal model heritability was determined by:

$$h^{2}=\frac{\sigma_{a}^{2}}{\sigma_{p}^{2}}$$

where $\sigma_{a}^{2}$ is the animal variance. To be defined as heritable, the inclusion of sire or animal in the final model had to be significant based on a likelihood ratio test *P*-value of <0.05, and the heritability estimate generated had to exceed the 95% confidence interval (1.96 × standard error). For this reason, results pertaining to sesamoid bone fracture, distal limb fracture, superficial digital flexor tendon injury and degenerative joint disease are not shown. Genetic correlations were determined by generation of appropriate bivariate sire linear models without fixed effects.

## Results

### Pedigree

The complete pedigree file contained 12,169 horse identities. The number of generations per horse in the pedigree ranged from 0 to 8 with a mean of 2.3 and a median of 2. The 5062 phenotyped horses had 1296 sires (mean 3.9, median 2, range 1–167 offspring per sire) and 4562 dams (mean 1.1, median 1, range 1–5 offspring per dam).

### Risk factor analysis

There were a total of 5062 horses represented in this study. Of these, 675 horse records included a diagnosis of fracture (13%) and 508 included a diagnosis of osteoarthritis (10%). Suspensory ligament injury (523) and tendon injury (952) were diagnosed in 10% and 19% of records, respectively. Of the 15 potential risk factors for each condition, one was found to be associated with fracture, one with osteoarthritis, three with suspensory ligament injury, and four with tendon injury (Table 1). Most conditions were associated with variables relating to accumulation of time at risk. Originating from Australasia compared with Europe was associated with reduced odds of suspensory ligament injury, but was also a risk factor for tendon injury. Originating from North America as opposed to Europe was also associated with reduced odds of suspensory ligament injury, and originating from ‘any other continent’ as opposed to Europe was identified as a risk factor for tendon injury. Decreasing intensity of racing, i.e. longer periods between adjacent races, was associated with increased odds of tendon injury.

### Heritability estimation

Inclusion of sire or animal as a random effect significantly improved the fit of most models (with the exception of osteoarthritis models), suggesting significant heritability (Table 2). Fracture heritability estimates ranged from 0.03 to 0.11, osteoarthritis from 0.01 to 0.15, suspensory ligament injury from 0.05 to 0.17 and tendon injury from 0.09 to 0.20 (Table 3). In all conditions, the largest heritability estimate was produced using the logistic sire model. In most conditions the smallest estimate was produced from the linear animal model, with the exception of tendon injury, for which the smallest estimate was produced by the logistic animal model. Sire models produced larger heritability estimates compared with their animal model equivalents in all conditions except tendon injury.

### Genetic correlations

Significant positive genetic correlations were found between fracture and osteoarthritis and fracture and suspensory ligament injury, as described in Table 4. All other genetic correlations investigated were not statistically significant.

## Discussion

This paper reports the results of a study in which the primary focus was to establish benchmark values for the heritability of important MSC in a Hong Kong racing Thoroughbred population. The limited size and variable specificity of free text ‘diagnoses’ entered into the HKJC health records may have resulted in a degree of misclassification bias, which could have limited the study validity. However, the procedures for content analysis detailed in Lam et al. (2007) proved to be reliable, and were adapted for use in this study. Some power may have been lost due to insufficient case numbers to model more specific conditions, e.g. sesamoid bone fracture. Additionally, caution must be used in interpretation of these results, as a degree of autoregression in the data was unavoidable, due to the likely career-ending nature of serious MSC. An analysis of the autoregression was not possible as text records did not typically contain information on whether the medical grounds for retirement were continuations of conditions recorded in OVE records, or constituted ‘new’ diagnoses.

The variables found to be associated with each condition in this study are by no means intended to be an exhaustive list of environmental risk factors, but are included for optimal model building and to make the best use of the available data. A number of previous studies have demonstrated an increased likelihood of MSC with increasing length of career, or time spent at risk (Bailey et al., 1998; Ely et al., 2004; Cogger et al., 2006; Boden et al., 2007; Jacklin and Wright, 2012). In the current study, longer career length was found to be associated with the risk of tendon injury. This variable is most likely describing time at risk.

Career history has been found to be associated with certain injuries in previous studies (Kasashima et al., 2004; Jacklin and Wright, 2012). A positive association between increasing intensity of racing, or number of starts, and the risk of injury have previously been demonstrated (Estberg et al., 1996; Verheyen et al., 2006; Boden et al., 2007; Anthenill et al., 2010). However, other studies failed to demonstrate this association, or demonstrated a negative association (Cohen et al., 2000; Parkin et al., 2004b; Perkins et al., 2005). The number of races run in the career of each horse was found to be negatively associated with the risk of tendon or suspensory ligament injury in this study. The apparent protective effect of running more races may be due to musculoskeletal tissue remodelling, leading to tendons and ligaments which are more resistant to damage, or may simply be an example of the ‘healthy horse’ effect where healthier horses are able to race more frequently.

Both fracture and osteoarthritis risk increased marginally with each subsequent cohort of horses, described by the year of retirement and year of birth variables. The precise reason for these associations is unknown. This may reflect a real, and therefore concerning rise in incidence, however, it may be that changes in the veterinary personnel, management of horses or the recording of health information over the years in Hong Kong, resulted in improved reporting of these conditions in later years.

The continent from which a horse originated was found to be associated with the risk of suspensory ligament and tendon injury. However, the nature of the association varied between conditions. For suspensory ligament injury, horses originating from North America or Australasia were at a reduced risk compared to those from Europe. For tendon injury, horses originating from Australasia and ‘other’ continents (Asia, Africa or South America) were at increased risk compared with those from Europe, and horses originating from North America were at reduced risk compared to those from Europe. These differences are difficult to explain. However, Thoroughbred populations from different continents most likely represent different genetic groups, and it is also likely that differing management of horses before importation could have had lasting effects on their risks of injury. Further analysis of these associations is outside the scope of this paper.

The heritability estimates for fracture, osteoarthritis and suspensory ligament injury reported here are small to moderate in size, with large standard errors. No previous estimates of the heritability of these conditions in the horse could be found in the literature. Osteochondrosis dissecans (OCD), a developmental orthopaedic condition common in horses, has been studied in the past and found to have a heritability in the range 0.03–0.32, based on various methods and in different horse breeds (Philipsson et al., 1993; Pieramati et al., 2003; van Grevenhof et al., 2009; Jonsson et al., 2011). OCD is known to be a risk factor for osteoarthritis, although due to the nature of the veterinary records in this study it was not possible to investigate OCD heritability specifically.

The estimate of heritability of tendon injury was found to be large for a binary trait (0.09–0.20), which is similar to an estimate of the heritability of superficial digital flexor tendon (SDFT) injury found using a Bayesian modelling approach in a Japanese racing cohort by Oki et al. (2008). It is likely that the great majority of tendon injuries in this study involved the SDFT. However, records often omitted this level of detail, so it is difficult to compare tendon injury heritability directly from this study with SDFT injury heritability. The magnitude of heritability estimates reported here suggest that progress to reduce disease incidence in this population could be made through targeted breeding strategies based on selection for estimated breeding values for each condition. Significant positive genetic correlations between fracture, osteoarthritis and suspensory ligament injury suggest that efforts could be focused upon whichever of these conditions is most easily phenotyped, and progress in the reduction of genetic risk of the correlated condition might occur concurrently.

Sire models account for the non-independence of individuals in the pedigree through generating half-sibling groups. These models assume founders are unrelated, and that mating occurs at random. Both of these assumptions may be incorrect in the Thoroughbred population. However, sire models have proven useful in situations in which there was thought to be negligible variance attributable to grouping by a common dam, in which pedigree information suffered from a large proportion of missing information beyond the parent level, in which computational difficulties preclude use of animal models, or in which there are few records per individual (Matos et al., 1997; Stock and Distl, 2006; Gilmour et al., 2009; BHA, 2012). Animal models, in comparison, take into account all types of relationship between individuals, are computationally more intensive, and can suffer from sparse information per individual. As a consequence, animal models will frequently not converge, whereas the equivalent sire model will produce a statistically stable model. Both types of model were included here for comparison. The majority of heritability estimates using sire models exceeded those from animal models, which is likely to reflect the data structure, where sires are unequally represented in terms of number of offspring (Matos et al., 1997; Weideman et al., 2004; Stock and Distl, 2006).

Binary data can be modelled with a linear mixed model approach, but may suffer from a loss of precision if the observed 0/1 scale is used. To avoid this loss of precision, logistic regression can be used, with which the linear function of the explanatory variables is related to the mean of the probability distribution of the response variable by a link function derived from the probability of the condition in the population (Matos et al., 1997; van Grevenhof et al., 2009; Vazquez et al., 2009; Lewis et al., 2011). In almost all cases, logistic heritability estimates exceeded their linear model equivalents, suggesting that the nature of the conditions studied would be better represented by a continuous or polychotomous scale of measurement. However, it is worth noting that all estimates of heritability within each trait, with the possible exception of logistic sire model estimates, were not significantly different from each other.

## Conclusions

This study has found that the risks of fracture, osteoarthritis, suspensory ligament injury and tendon injury have significant heritable components in this Hong Kong study population, which suggests that selective breeding approaches may be successful in reducing genetic risk in future. The data generated will also serve as a benchmark for future studies, and could be used as a comparator to studies aimed at estimating the heritability of disease-associated markers in different racing jurisdictions or Thoroughbred populations.

## Conflict of interest statement

None of the authors of this paper has a financial or personal relationship with other people or organisations that could inappropriately influence or bias the content of the paper.

## Figures and Tables

**Table 1:** Results of multivariable logistic regression models investigating risk factors for four musculoskeletal conditions in Hong Kong Thoroughbreds. These results refer to sire model analyses only. There were no differences in significance or direction of associations between risk factors and outcomes using animal models. Percentages are calculated by row.

Musculoskeletal condition	Risk factors	Total	Cases (%)	Controls (%)	LRT⁎*P*-value	Odds ratio (OR)	95% Confidence interval
Fracture	Year of birth (1985 to 2007)	5062	675 (13)	4387 (87)	<0.001	1.11	1.09–1.14

Osteoarthritis	Year of retirement (1995 to 2010)	5062	508 (10)	4554 (90)	<0.001	1.43	1.38–1.48

Suspensory ligament injury	Age at retirement (year)	5062	523 (10)	4539 (90)	<0.001	1.45	1.34–1.57
	Number of races run	5062	523 (10)	4539 (90)	<0.001	0.99	0.98–0.99
	Continent group^¥^: *1*	3189	355 (11)	2834 (89)	<0.001	1 (REF)	
	*2*	1196	114 (10)	1082 (90)		0.71	0.55–0.90
	*3*	585	48 (8)	537 (92)		0.61	0.43–0.85
	*4*	92	6 (7)	86 (93)		0.55	0.23–1.33

Tendon injury	Intensity of racing (weeks between races)	5062	952 (19)	4110 (81)	<0.001	1.04	1.02–1.05
	Number of races run	5062	952 (19)	4110 (81)	<0.001	0.95	0.94–0.96
	Continent group^a^: 1	3189	541 (17)	2648 (83)	0.0029	1 (REF)	
	2	1196	302 (25)	894 (75)		1.66	1.40–1.98
	3	585	80 (14)	505 (86)		0.75	0.57–0.98
	4	92	29 (32)	63 (68)		2.28	1.42–3.67
	Length of career (year)	5062	952 (19)	4110 (81)	<0.001	1.41	1.25–1.58

⁎ Likelihood ratio test.

a Continent group: 1, Europe; 2, Australasia; 3, North America; 4, other; REF indicates the reference group to which the other levels of a categorical variable were compared.

**Table 2:** Variance at the sire or animal level, in linear and logistic models of each condition. Figures in parentheses are *P*-values of likelihood ratio tests between each final model, and the corresponding model with the random effect (only) omitted.

	Linear sire model	Logistic sire model	Linear animal model	Logistic animal model
Fracture	0.0011 (0.02)	0.0919 (<0.01)	0.0032 (0.02)	0.1622 (<0.01)
Osteoarthritis	0.0007 (0.10)	0.1248 (<0.01)	0.0006 (n/a)	0.1876 (<0.01)
Suspensory ligament injury	0.0014 (0.01)	0.1463 (<0.01)	0.0043 (0.02)	0.2413 (<0.01)
Tendon injury	0.0046 (<0.01)	0.1756 (<0.01)	0.0209 (<0.01)	0.3169 (<0.01)

**Table 3:** Heritability estimates of four musculoskeletal conditions derived from linear and logistic sire and animal models.

	*h*^2^ (standard error)			
	Linear sire model	Logistic sire model	Linear animal model	Logistic animal model
Fracture	0.040 (0.025)	0.109 (0.063)	0.028 (0.019)	0.047 (0.032)
Osteoarthritis	0.034 (0.025)	0.146 (0.083)	0.007 (0.015)	0.054 (0.041)
Suspensory ligament injury	0.060 (0.030)	0.170 (0.091)	0.047 (0.024)	0.068 (0.042)
Tendon injury	0.127 (0.039)	0.203 (0.069)	0.142 (0.035)	0.088 (0.029)

**Table 4:** Statistically significant genetic correlations between musculoskeletal conditions.

	*r*_g_	Standard error	*P*-value
Fracture and osteoarthritis	0.85	0.13	<0.001
Fracture and suspensory ligament injury	0.59	0.29	<0.05

## Footnotes

1 See: http://pedigreeinfo.com.
